# Clinical effects of glucagon-like peptide-1 receptor agonist in type 2 diabetes with low body mass index: findings from large-scale emulated target trials

**DOI:** 10.7150/ijms.130295

**Published:** 2026-03-30

**Authors:** Ming-Hsien Tsai, Yu-Wei Fang, Yu-Han Tsai, Meng-Ting Chen, Kuo-Cheng Lu, Chien-Lin Lu

**Affiliations:** 1 Division of Nephrology, Department of Internal Medicine, Shin-Kong Wu Ho-Su Memorial Hospital, Taipei, Taiwan.; 2 Department of Digital Medicine, Shin-Kong Wu Ho-Su Memorial Hospital, Taipei, Taiwan.; 3 School of Medicine, College of Medicine, Fu Jen Catholic University, New Taipei City, Taiwan.; 4 Department of Medicine, Chang Gung University, Taoyuan, Taiwan; 5 Division of Nephrology, Department of Medicine, Taipei Tzu Chi Hospital, Buddhist Tzu Chi Medical Foundation, New Taipei City, Taiwan.; 6 Division of Nephrology, Department of Internal Medicine, Fu Jen Catholic University Hospital, Fu Jen Catholic University, New Taipei City, Taiwan.

**Keywords:** Cardiovascular outcomes, GLP-1 receptor agonists, kidney outcomes, Type 2 diabetes

## Abstract

**Aims:**

Chronic kidney disease (CKD) is a common and serious complication of type 2 diabetes, yet the effectiveness of glucagon-like peptide-1 receptor agonists (GLP-1 RAs) in non-obese or mildly overweight individuals remains uncertain. This study evaluated renal, cardiovascular, and systemic outcomes associated with GLP-1 RA therapy in adults with type 2 diabetes and body mass index (BMI) ≤ 30 kg/m².

**Materials and Methods:**

We conducted a real-world, target trial emulation, retrospective cohort study using the TriNetX US Collaborative Network. Adults with type 2 diabetes and BMI ≤30 kg/m² initiating GLP-1 RAs or dipeptidyl peptidase-4 inhibitors (DPP-4i) between 2016 and 2023 were identified. After exclusions, 23,103 GLP-1 RA and 44,156 DPP-4i users remained; 1:1 propensity score matching yielded two balanced cohorts of 20,928 patients. Outcomes—including major adverse kidney events (MAKE), progression to dialysis, cardiovascular events, hospitalization, and sepsis—were assessed over up to four years. Cox regression and Kaplan-Meier analyses estimated hazard ratios.

**Results:**

GLP-1 RA initiation was associated with lower risks of MAKE (14.8% vs. 16.8%; HR 0.93, p = 0.005) and progression to dialysis (HR 0.78, p < 0.001). Cardiovascular outcomes and all-cause mortality were similar between groups. GLP-1 RAs significantly reduced hospitalization (HR 0.84, p < 0.001) and sepsis (HR 0.88, p = 0.001). Benefits were consistent across BMI strata and clinical subgroups, with no evidence of effect modification.

**Conclusions:**

In adults with type 2 diabetes and BMI ≤ 30 kg/m², GLP-1 RAs confer clinically meaningful kidney protection and reduce hospitalization and sepsis, despite neutral cardiovascular effects. These findings support the use of GLP-1 RAs in non-obese or mildly overweight diabetic populations.

## Introduction

Chronic kidney disease (CKD) is a major complication of type 2 diabetes and affects roughly one-quarter to one-third of patients in several Asian regions. Reported prevalence ranges from 24-35% in Thailand and India to about 31-33% in China, and studies from South Asia describe a rapid decline in kidney function once CKD develops 1-3. A substantial portion of this burden occurs in individuals with relatively low body mass index (BMI). This “lean diabetes” pattern—common in many Asian countries—is characterized by limited adiposity but pronounced metabolic risk and is linked to earlier onset of CKD and faster loss of eGFR 4, 5. Similar lower-BMI phenotypes are now described outside Asia as well, indicating that many patients with type 2 diabetes differ from the predominantly overweight profiles represented in most Western trials.

Glucagon-like peptide-1 receptor agonists (GLP-1 RAs) have been shown in large cardiovascular outcome trials to reduce major adverse cardiovascular events (MACE) by roughly 13-15% in high-risk type 2 diabetes populations, as demonstrated in LEADER, SUSTAIN-6, and REWIND 6-8. Renal endpoints from these trials and related meta-analyses consistently show reduced progression of albuminuria (approximately 21-26%) and modest protection against composite kidney outcomes, including sustained eGFR decline and kidney failure 9-11. Experimental and translational work suggests several kidney-specific actions of GLP-1 RAs, including effects on tubular sodium handling, tubuloglomerular feedback, and pathways related to renal inflammation and fibrosis, many of which appear to occur independently of weight change 12-15.

Whether these kidney benefits extend to individuals with lower BMI is less certain. Most randomized trials included patients who were overweight or obese, and individuals with BMI below 30 kg/m² constitute a minority of participants. Observational data from Taiwan suggest that the cardiovascular effects of GLP-1 RAs may differ by BMI, with clearer benefit at BMI ≥ 25 kg/m², while kidney outcomes appear less affected by adiposity 16. Meta-analyses also indicate potential variation in cardiovascular response by BMI but little evidence that renal effects differ substantially across weight categories 17-19. These observations raise an important question regarding the effectiveness of GLP-1 RAs in the growing number of patients with type 2 diabetes who are non-obese or only mildly overweight.

To examine this issue, we conducted a real-world, target trial emulation comparing GLP-1 RAs with dipeptidyl peptidase-4 inhibitors (DPP-4i) in adults with type 2 diabetes and BMI ≤ 30 kg/m². Using a new-user, active-comparator design with broad covariate adjustment through propensity score matching, we evaluated major adverse kidney events (MAKE), progression to dialysis, cardiovascular outcomes, hospitalization, and sepsis. Our aim was to determine whether GLP-1 RAs provide kidney and systemic benefits in patients who fall outside the typical BMI profile of prior randomized trials.

## Materials and Methods

### Data sources

TriNetX is a global health research platform that accelerates medical discoveries by securely connecting healthcare organizations, pharma companies, and academic researchers. It aggregates and anonymizes real-world patient data from electronic healthy records (EHRs) and insurance claims in a continuously updated, cloud-based database. Researchers use TriNetX's intuitive tools to define patient cohorts, quickly assess study feasibility, and conduct robust analyses using real data. The platform streamlines clinical trial recruitment, optimizes study design, and offers advanced analytics to visualize treatment patterns and predict health trends. Its specialized networks foster collaboration across institutions, helping tackle challenges like rare disease research. With strong privacy measures compliant with Health Insurance Portability and Accountability Act and General Data Protection Regulation*,* TriNetX enables comprehensive, compliant research and drives meaningful advancements in patient care and evidence-based medicine.

### Ethics statement

This study was approved by the Institutional Review Board of Shin Kong Wu Ho-Su Memorial Hospital (IRB number: 20250907R) and the patients' informed consent was waived because as TriNetX provides access to a de-identified database for patient selection.

### Study design and study population

**Figure 1:**
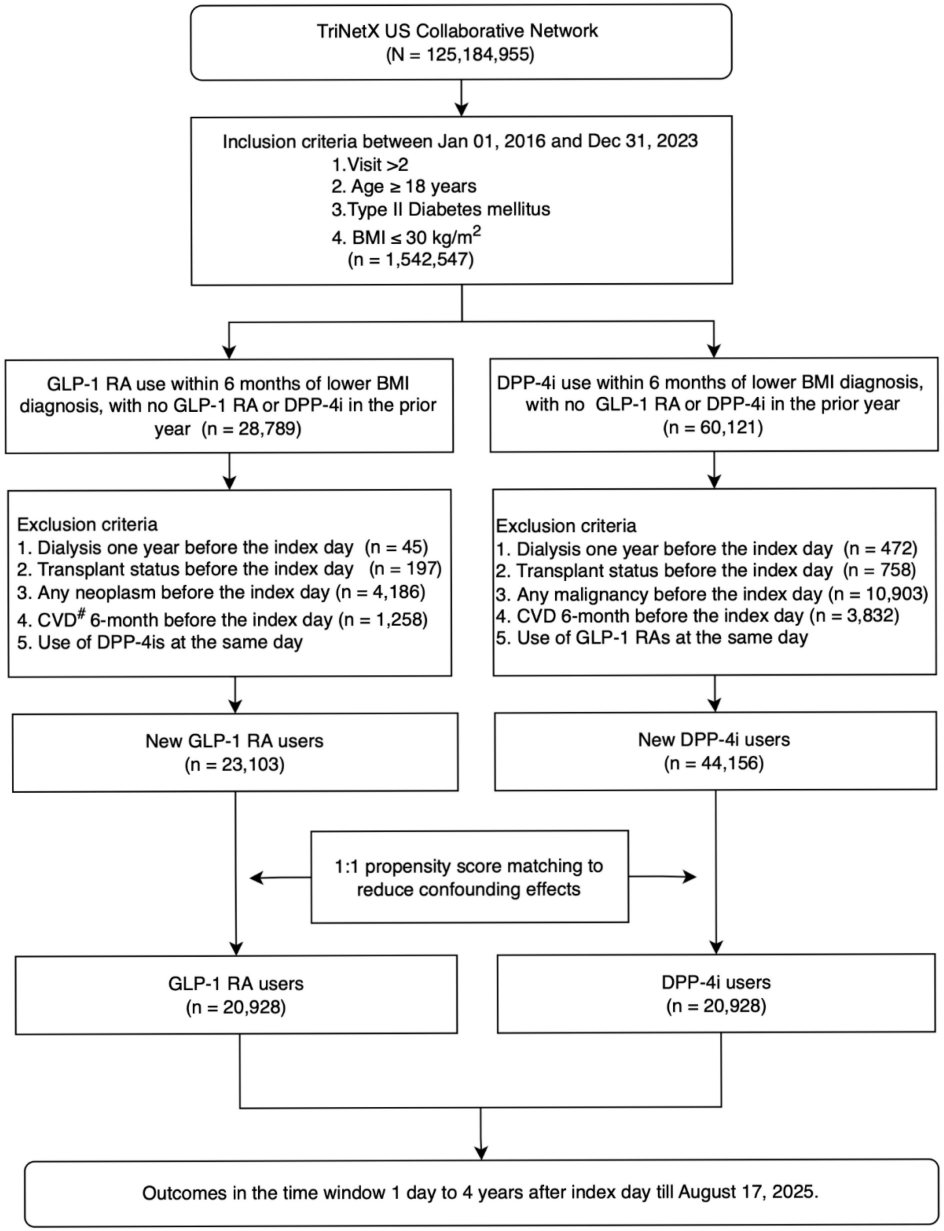
** The schema of patient enrollment in the study.** Abbreviation: eGFR, estimated glomerular filtration rate.

This retrospective cohort study, as illustrated in Figure 1, utilized a new-user, intention-to-treat, and active-comparator design to evaluate outcomes associated with GLP-1 RA and DPP-4 inhibitor (DPP-4i) use among adults with type 2 diabetes mellitus based on target trial emulation guidelines 20. The analysis was based on the TriNetX US Collaborative Network, comprising a large population of 125,184,955 individuals. Patients were eligible if they were aged 18 years or older, had a diagnosis of type 2 diabetes, and a recorded BMI of 30 kg/m² or below, and had at least two clinical encounters recorded in the database between January 1, 2016, and December 31, 2023. The requirement for at least two visits was applied as a data-density criterion to ensure adequate baseline clinical information and active engagement with the healthcare system, and was not used as a requirement for survival until the end of the study period. This criterion was applied before cohort assignment and equally to both treatment groups and therefore was unlikely to introduce differential selection-related bias or immortal time bias. The index date was defined as the date of each patient's first prescription of either a GLP-1 RA or a DPP-4i within 6 months after documentation of BMI ≤ 30 kg/m^2^, provided that no use of either drug class occurred in the preceding year. BMI ≤ 30 kg/m^2^ was defined based on the most recent recorded BMI measurement prior to treatment initiation and was not required to be sustained over time. Longitudinal weight trajectories before cohort entry could not be fully characterized due to platform-level data constraints.

Follow-up for each patient began at the index date and continued until the occurrence of an outcome event, loss to follow-up, or the end of the study period. Two mutually exclusive new-user cohorts were identified: GLP-1 RA users (n = 28,789) and DPP-4i users (n = 60,121), from which patients meeting specific exclusion criteria—such as history of dialysis, transplant, neoplasms or malignancy, recent cardiovascular disease, or concurrent use of the comparator drug class—were removed. This process yielded 23,103 new GLP-1 RA users and 44,156 new DPP-4i users.

To control for confounding, 1:1 propensity score matching (PSM) was performed, resulting in two balanced groups, each comprising 20,928 patients. Outcomes were assessed in a time window from 1 day up to 4 years following the index date, through August 17, 2025. Cohort definitions are described in A Supplementary Method S1.

### Covariates

Baseline covariates included demographic variables, comorbidities, medication use, and laboratory measurements recorded within 1 year before the index date: The baseline characteristics assessed in this study encompassed a diverse range of demographic, clinical, and laboratory parameters. Demographic factors included BMI, age at index, sex distribution (male), and racial/ethnic composition (White, African American, Asian, and other races). Furthermore, comorbidity data were collected for hypertensive diseases, dyslipidemia, ischemic heart diseases, disorders of thyroid gland, other anxiety disorders, nicotine dependence, hepatic failure, cerebrovascular diseases, fatty liver, gout, cardiomyopathy, pulmonary heart disease, atherosclerosis of native arteries of the extremities, liver cirrhosis, and chronic rheumatic heart diseases. In addition, medication use was documented, including insulin, biguanides, sulfonylureas, sodium-glucose co-transporter 2 inhibitors (SGLT2i), thiazolidinediones, statins, fibrates, renin-angiotensin system blockade (RASB), beta-blockers, calcium channel blockers, antithrombotic agents, nonsteroidal anti-inflammatory drugs (NSAIDs), immunosuppressants, and immunostimulants. Moreover, comprehensive laboratory measurements were obtained, encompassing estimated glomerular filtration rate (eGFR), urea nitrogen, sodium, potassium, calcium, phosphate, albumin, hemoglobin, hemoglobin A1c, alanine aminotransferase (ALT), aspartate aminotransferase (AST), alkaline phosphatase, low-density lipoprotein (LDL), high-density lipoprotein (HDL), triglycerides, C-reactive protein, and urine albumin-to-creatinine ratio (UACR). eGFR was calculated using the 2021 Chronic Kidney Disease Epidemiology Collaboration (CKD-EPI) equation without inclusion of a race coefficient 21. The codes for the baseline covariates are shown in Supplementary Method S1.

### Study outcomes

The study focused on several key clinical outcomes that occurred within a time window of three to four years after the index event, with follow-up data collected until August 17, 2025. The primary outcome assessed was MAKE, which included acute kidney injury, end-stage kidney disease, initiation of dialysis, and death. AKI was identified using diagnosis codes for acute kidney failure (ICD-10-CM N17). ESKD on dialysis was defined using diagnosis codes for end-stage renal disease (ICD-10-CM N18.6) and dependence on renal dialysis (ICD-10-CM Z99.2), as well as dialysis-related procedure codes. In addition to the composite primary outcome, several secondary outcomes were evaluated to capture broader clinical implications. These included individual kidney-related outcomes such as entering dialysis and acute kidney injury, as well as major cardiovascular and health outcomes such as all-cause mortality, acute myocardial infarction (AMI), heart failure, stroke, hospitalization for any cause, and sepsis. Together, these outcomes provided a comprehensive view of renal, cardiovascular, and overall health trajectories among patients over the follow-up period. The outcome codes are listed in Supplementary Method S1.

### Sensitivity analysis

To ensure our findings were robust, we conducted four sensitivity analyses to address potential limitations and confirm the consistency of our results. First, we applied different propensity score matching (PSM) models, each adjusted for a range of confounding factors, to minimize bias and isolate the effects under investigation. Second, we assessed the risk across multiple time intervals to address possible violations of the proportional hazard assumption, ensuring the validity of our conclusions over time. Third, we performed analyses using 3-month lags after the index date to test the stability of our results with respect to different latency periods. Finally, the E-value was calculated for each result as a sensitivity analysis to quantify the minimal strength of association that an unmeasured confounder would need to have with both the exposure and the outcome to fully explain away the observed association, after accounting for the measured covariates.^22^ These approaches collectively strengthened the credibility and generalizability of our findings.

### Statistical analysis

Categorical variables were presented as counts and percentages, while numerical variables were reported as means with standard deviations. Group differences were assessed using standardized mean differences (SMD) 23, considering values below 0.1 as an indication of well-balanced groups. To minimize baseline confounding and improve comparability, PSM was implemented, resulting in two matched cohorts 24. This approach allowed for more accurate evaluation of treatment effects by ensuring groups were similar across baseline demographic, clinical, and laboratory characteristics. Propensity scores were derived utilizing logistic regression and subsequently matched through a greedy nearest-neighbor approach with a 0.1 caliper, aimed at achieving equilibrium among treatment groups for the purpose of analysis. In the process of PSM, the age at the index date was regarded as a continuous variable, whereas sex, race, lifestyle attributes, comorbid conditions, and pharmacological interventions were classified as categorical (present/absent). Laboratory metrics were deemed present if they were obtainable, or absent if not recorded within the designated timeframe.

Cox proportional hazards regression was used to calculate hazard ratios (HRs) for comparing clinical outcome risks between groups. The proportional hazards assumption was evaluated using the generalized Schoenfeld method within TriNetX. Event-free probabilities were estimated with Kaplan-Meier curves, providing a visual comparison of outcome incidence over time. In the present investigation, we examined 9 hypotheses while anticipating a false discovery rate of 0.05. The Benjamini-Hochberg approach was employed to adjust the P values accordingly.

Subgroup analyses were performed, with all subgroups being re-matched through propensity score matching (PSM) based on initial characteristics to guarantee well-balanced and comparable pairs. Because interaction testing is not directly supported in the TriNetX interface, subgroup heterogeneity was assessed using Cochran's Q test and I^2^ statistics in a random-effects model 25. The p-value for heterogeneity was calculated to determine whether the influence of SGLT2 inhibitors on clinical outcomes demonstrated significant variability across different subgroups.

All statistical analysis was conducted in the TriNetX platform. A two-sided *P*-value of < 0.05 was considered significant. R software version 4.4.2 (Free Software Foundation Inc.), with the Forestploter and ggplot2 packages, was used to create the figures in this study.

## Results

### Baseline characteristics of patients

Figure 1 summarizes cohort selection. Among 67,259 adults with type 2 diabetes and BMI ≤ 30 kg/m², exclusions included prior GLP-1 RA or DPP4i exposure, kidney failure, transplantation, malignancy, recent cardiovascular events, and concurrent comparator use, yielding 23,103 GLP-1 RA initiators and 44,156 DPP4i initiators before matching. After 1:1 propensity score matching, two balanced cohorts of 20,928 patients each were formed. Baseline characteristics were well aligned, with all standardized mean differences < 0.1 (Table 1). Mean age was approximately 61 years, and 52% were male. Comorbidities, laboratory values, and concomitant medications were highly comparable between groups. The study structure, exposure definition, and follow-up scheme followed a prespecified target trial framework (Table S1), and the overall design—including eligibility windows, assignment, and index date construction—is shown in Figure S1.

Laboratory data were incompletely available for a substantial proportion of patients. In particular, urine albumin-to-creatinine ratio (uACR) measurements were missing in most individuals, with missing rates of 95.1% in the GLP-1 RA group and 96.5% in the DPP-4i group after matching (Supplementary Method S1). Accordingly, uACR was not incorporated into the primary propensity score model and was interpreted cautiously in subgroup analyses.

### Major adverse kidney outcomes

Kaplan-Meier curves demonstrated significantly higher event-free survival among GLP-1 RA users for MAKE (p = 0.005) and ESKD requiring dialysis (p < 0.001) (Figure S2A-B). No differences were observed in all-cause mortality (p = 0.818) or acute kidney injury (AKI; p = 0.222) (Figure S2C-D). GLP-1 RA use was also associated with a lower risk of sepsis (p = 0.001) (Figure S2E). As shown in Table 2, MAKE occurred in 14.8% of GLP-1 RA users versus 16.8% of DPP4i users (HR 0.93, 95% CI 0.89-0.98). The risk of ESKD on dialysis was significantly lower with GLP-1 RA (HR 0.78, 95% CI 0.70-0.87), whereas risks of AKI, acute myocardial infarction, and mortality were similar between groups. Associations for MAKE and dialysis remained significant after false discovery rate correction, and E-value analysis suggested that the observed associations for MAKE (1.36) and dialysis (1.88) would require a confounder with at least moderate strength of association with both treatment and outcome to be fully explained away. Subsequent to the implementation of statistical adjustments for multiple comparisons, those levels of significance were preserved.

### Other clinical outcomes

In secondary analyses, GLP-1 RA users had significantly lower cumulative incidence of hospitalization (p < 0.001) and sepsis (p = 0.001), while event-free survival for acute myocardial infarction, heart failure, and stroke did not differ between groups (Figure S3). Hazard ratios from Table 2 were consistent with the KM findings: hospitalization (HR 0.84, p < 0.001) and sepsis (HR 0.88, p = 0.001) were significantly reduced, whereas cardiovascular outcomes and mortality were neutral (HRs ≈ 1.00). Similar robustness was observed for secondary outcomes, including hospitalization (E-value = 1.67) and sepsis (E-value = 1.53).

### BMI-stratified analyses

Associations were generally consistent across BMI levels (Table 3). Among patients with BMI 25-30 kg/m², GLP-1 RA use was associated with reduced risks of entering dialysis (HR 0.87, 95% CI 0.74-1.01), AKI (HR 0.92, 95% CI 0.84-0.99), hospitalization (HR 0.84, 95% CI 0.79-0.86), and sepsis (HR 0.81, 95% CI 0.72-0.90). In those with BMI < 25 kg/m², GLP-1 RA therapy similarly lowered risks of entering dialysis (HR 0.72, 95% CI 0.54-0.95), hospitalization (HR 0.81, 95% CI 0.73-0.90), and sepsis (HR 0.80, 95% CI 0.65-0.98). Mortality and cardiovascular outcomes remained neutral across all BMI strata. Overall, renal and systemic benefits were preserved irrespective of BMI category.

### Subgroup analysis

**Figure 2:**
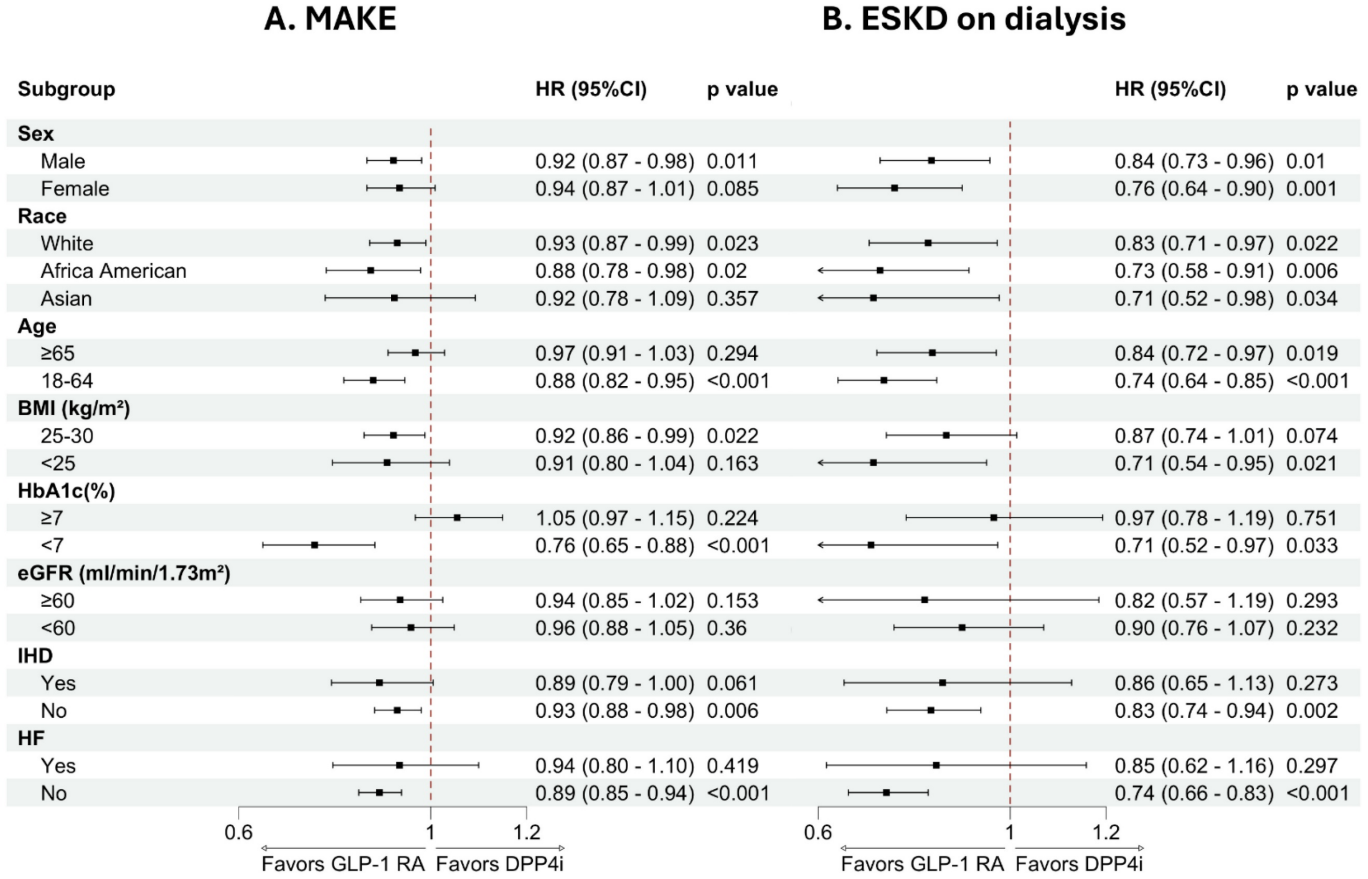
** Subgroup analyses of MAKE and ESKD on dialysis comparing GLP-1 RA and DPP-4 inhibitor users.** (A) Hazard ratios for MAKE and (B) ESKD requiring dialysis across key clinical subgroups. Points represent hazard ratios with 95% CIs; the dashed line denotes HR = 1.0. Values <1.0 favor GLP-1 RA over DPP-4i. Abbreviations: MAKE, major adverse kidney events; ESKD, end-stage kidney disease; eGFR, estimated glomerular filtration rate; HF, heart failure; GLP-1 RA, glucagon-like peptide-1 receptor agonist; DPP-4i, dipeptidyl peptidase-4 inhibitor.

Subgroup analyses in Figure 2 showed that the point estimates consistently favored GLP-1 RA over DPP-4i for both MAKE and dialysis across most demographic and clinical strata. Statistically significant reductions in MAKE were observed primarily among males, younger individuals (18-64 years), and those with BMI 25-30 kg/m^2^. Race-specific analyses demonstrated significant benefit in White and African American patients. As shown in Figure S4, although not all subgroups reached statistical significance, the hazard ratios were generally below 1.0, and no significant treatment-subgroup interactions were detected.

For the dialysis outcome (Figure S5), subgroup analyses demonstrated a similar overall pattern of risk reduction across strata. Statistically significant associations were observed in both sexes, across racial groups, and in both age categories. Reductions in dialysis risk were particularly evident among individuals with BMI <25 kg/m^2^ and those without heart failure. For other subgroups, the direction of effect consistently favored GLP-1 RA, although statistical significance was not uniformly observed.

### Sensitivity analysis

Findings were robust across all sensitivity analyses. Results were consistent across multiple propensity score models (Table S2), across 1-, 2-, and 3-year follow-up windows (Table S3), and after applying a 3-month lag to minimize reverse causation (Table S4). The E-values for the significant outcomes (1.36-1.88) indicate the minimum strength of association that an unmeasured confounder would need to have with both treatment and outcome to fully account for the observed associations (Table 2). Although residual confounding cannot be excluded, the consistency of findings across multiple outcomes supports the robustness of the observed associations.

## Discussion

In this large real-world cohort of adults with type 2 diabetes and BMI ≤30 kg/m², initiation of GLP-1 receptor agonists, compared with DPP-4 inhibitors, was associated with a modest but steady reduction in MAKE and a more pronounced reduction in progression to dialysis. These kidney benefits were accompanied by lower risks of hospitalization and sepsis, whereas cardiovascular outcomes and overall mortality did not differ between groups. The preservation of these benefits across BMI categories (<25 and 25-30 kg/m²) and across multiple clinical subgroups suggests that GLP-1 RA therapy may offer meaningful kidney and systemic protection in non-obese or mildly overweight individuals with type 2 diabetes, even in the absence of measurable cardiovascular advantages.

Our results parallel observations from a recent nationwide cohort in Taiwan using the Chang Gung Research Database, in which GLP-1 RA therapy was linked to substantial reductions in MACE, cardiovascular death, and heart failure hospitalization among patients with BMI ≥ 25 kg/m², but not in those with BMI < 25 kg/m² 16. In that study, kidney outcomes—defined by major eGFR decline or dialysis—were comparable across BMI strata. A similar pattern emerged in our analysis: cardiovascular outcomes remained neutral within the BMI-restricted population, whereas kidney protection was evident. By applying a target trial emulation and excluding individuals with baseline kidney failure or early outcome events, the present study provides further evidence that the nephroprotective properties of GLP-1 RAs are maintained even when cardiovascular effects are less pronounced. This is consistent with several recent meta-analyses showing that reductions in MACE, cardiovascular death, and myocardial infarction are concentrated in patients with BMI ≥ 25 kg/m², with considerably weaker effects in those with lower BMI 17-19. These findings support a biologically plausible mechanism in which obesity-driven inflammation and insulin resistance amplify cardiovascular responsiveness to GLP-1 RA therapy 26-30.

The kidney outcomes in our study align with findings from cardiovascular outcome trials such as LEADER, SUSTAIN-6, and REWIND, in which liraglutide, semaglutide, and dulaglutide lowered the risk of composite kidney outcomes by hazard ratios of roughly 0.64-0.85, driven mainly by reduced macroalbuminuria, with additional moderate slowing of eGFR decline and some indication of reduced kidney replacement therapy 31-34. Pooled analyses show that these renal benefits are stable across BMI categories and other clinical subgroups 35-38. Several biological processes may underlie the largely BMI-independent nature of kidney protection. Experimental studies demonstrate that GLP-1 signaling induces natriuresis through inhibition of the proximal tubular sodium-hydrogen exchanger NHE3 14, 39, improves intraglomerular hemodynamics by reducing hyperfiltration and strengthening tubuloglomerular feedback 15, 40, and acts directly on GLP-1 receptors expressed in renal tubular and vascular cells 14. Beyond these hemodynamic effects, GLP-1 RAs reduce renal inflammation, oxidative injury, and fibrosis via PKC-related pathways, PKA activation, and modulation of TGF-β/Smad signaling 41-43. These mechanisms do not rely on weight loss or changes in BMI and fit well with the kidney protection observed in our non-obese cohort, including individuals with BMI < 25 kg/m².

The cardiovascular results contrasted with the renal findings. Rates of myocardial infarction, heart failure, stroke, and mortality were similar between groups, and survival curves did not diverge. Large cardiovascular outcome trials have shown MACE reduction with GLP-1 RAs in high-risk populations 10, 19, 33, 44-46, and the Taiwanese cohort reported clear benefits among users with BMI ≥ 25 kg/m² 16. The lack of signal in our BMI-restricted cohort may reflect the influence of body composition on treatment response. Obesity accompanies marked insulin resistance, expanded visceral fat, and chronic low-grade inflammation—factors that contribute directly to endothelial dysfunction, atherosclerotic progression, and myocardial remodeling. GLP-1 RAs can counteract these abnormalities by improving insulin sensitivity in muscle and adipose tissue, reducing visceral fat, and lowering systemic inflammatory cytokines such as TNF-α and IL-6; these responses tend to be stronger in individuals with higher BMI 29, 47. This biological context helps explain why cardiovascular effects were muted in our non-obese population despite clear kidney benefits 48, 49. Evidence from SELECT and STEP-HFpEF further supports this interpretation, as semaglutide improved heart failure symptoms and reduced MACE in overweight and obese patients—including those without diabetes—highlighting the central role of adiposity in shaping therapeutic response 50-52. In a cohort with limited adipose burden and relatively lower inflammatory activity, the treatment window for detectable cardiovascular benefit may simply be narrower.

In addition, the restriction of the study population to individuals with BMI ≤30 kg/m^2^ may have contributed to the absence of a detectable mortality benefit. Compared with populations enrolled in major cardiovascular outcome trials, our cohort likely had a lower baseline cardiovascular risk, which reduces statistical power to detect differences in hard endpoints such as death. This design choice was intentional, as our primary aim was to address the evidence gap in non-obese and mildly overweight patients with type 2 diabetes, who have been underrepresented in prior GLP-1 RA trials. Nevertheless, this focus necessarily limits the generalizability of our findings to patients with higher BMI, and our results should not be interpreted as excluding potential mortality benefits in more obese or higher-risk populations.

Beyond kidney and cardiovascular outcomes, our study identified meaningful reductions in hospitalization and sepsis among GLP-1 RA users, with divergence of curves early in follow-up. Similar reductions in infection-related hospitalizations were reported in the Chang Gung cohort across BMI categories 16. Several biologic processes may contribute. Improved glycemic stability may reduce exposure to extreme hyperglycemia, which is known to compromise innate and adaptive immunity. Preclinical studies have shown that GLP-1 analogs blunt inflammatory signaling triggered by endotoxin exposure, reduce vascular oxidative stress, preserve myocardial performance, and improve survival in models of endotoxemia and sepsis 53, 54. GLP-1 RAs also exert broader immune effects, including suppression of inflammatory cytokines, promotion of M2 macrophage activity, and downregulation of NF-κB signaling 55, 56. Reduced macrophage infiltration in adipose tissue and lower systemic inflammatory markers have been documented as well 57. Although our observational design does not allow direct attribution of the reduced sepsis risk to any specific pathway, the pattern is consistent with a combination of metabolic stabilization, kidney preservation, and systemic anti-inflammatory effects.

Subgroup analyses provided further insight into the consistency of treatment effects across clinical strata. The direction of association for both MAKE and dialysis generally favored GLP-1 RA therapy across age, sex, race, BMI, glycemic control, and comorbidity subgroups, with no signal of harm in any stratum. Statistically significant reductions in MAKE were mainly observed among males, younger individuals, and those with BMI 25-30 kg/m^2^, whereas reductions in dialysis risk were evident across both sexes and age categories and were particularly apparent among individuals without heart failure. These findings suggest that the renal benefits of GLP-1 RAs are broadly applicable in non-obese or mildly overweight patients with type 2 diabetes, rather than being confined to a narrowly defined clinical phenotype. The use of DPP-4 inhibitors as an active comparator further contextualizes the findings: EXAMINE, TECOS, and other major trials have shown neutral cardiovascular and kidney effects for this class 58, 59, making it a suitable reference rather than a competing cardiorenal therapy.

Several limitations warrant attention. Although the cohort was diverse, the applicability of our findings to Asian populations—where lean diabetes phenotypes are common—remains uncertain due to differences in diet, genetics, and clinical care. At the same time, the deliberate restriction to individuals with BMI ≤ 30 kg/m² represents a strength, as non-obese patients remain underrepresented in global GLP-1 RA trials. However, BMI ≤3 0 kg/m² in this study was defined based on a single recorded measurement rather than on longitudinal weight trajectories. Consequently, we were unable to distinguish individuals with stable long-term non-obese status from those who may have recently transitioned from higher BMI categories. Such preceding weight trends may be associated with underlying disease severity or treatment indications and could contribute to residual confounding.

As with all observational studies, residual confounding cannot be eliminated; factors such as socioeconomic status, physical activity, and genetic markers were not fully captured. A limitation is the requirement for ≥2 visits across the study period, which may introduce selection-related bias or potential immortal time bias by excluding patients with limited follow-up. However, because this criterion was applied uniformly to both cohorts before cohort assignment and propensity score matching, any potential bias would be expected to be nondifferential between groups. Outcome definitions relied on diagnostic coding and laboratory data, which introduces the possibility of misclassification, though the use of eGFR-based criteria mitigates some of these concerns. In particular, ESKD was identified using diagnosis and procedure codes rather than registry-based confirmation, and prior studies have shown that the specificity and positive predictive value of billing codes for ESKD may be limited. Misclassification of long-term dialysis status therefore cannot be fully excluded. In addition, urine albumin-to-creatinine ratio (uACR) was available for only a small subset of patients, which may introduce selection bias and limits the interpretability of subgroup analyses involving albuminuria. We also evaluated GLP-1 receptor agonists as a class; differences among individual agents—including pharmacokinetic properties, potency, and cardiovascular profiles—could not be examined. Lastly, medication adherence, dose escalation, and treatment duration were not fully assessed. In addition, our analyses were conducted under an intention-to-treat framework, and we did not perform an as-treated or per-protocol analysis. While our study shows a kidney-protective signal, the calculated E-values were modest (1.36 for MAKE and 1.88 for dialysis), indicating that an unmeasured confounder associated with both treatment selection and outcome by these magnitudes could potentially explain the observed associations. The relatively small E-value for MAKE suggests that the observed effect may be sensitive to residual confounding. Potential unmeasured factors, such as duration of diabetes, smoking status, hypertension severity, socioeconomic status, or medication adherence, may have influenced both treatment selection and outcomes.

The E-value analysis should be interpreted as a test of robustness rather than evidence of causality, and the observed associations may therefore be vulnerable to residual confounding. Similar ranges of E-values have been reported in other large real-world studies of GLP-1 receptor agonists. For example, Radwan et al. reported E-values of 1.21 and 2.73 for mortality outcomes in a nationwide observational cohort 60. These comparisons indicate that the degree of unmeasured confounding required to negate the observed associations in the present study is comparable to that reported in prior pharmacoepidemiologic analyses. Accordingly, the modest E-values should be regarded as a limitation of the present study, and the results should be interpreted with appropriate caution. In addition, treatment discontinuation and changes in exposure over time in routine clinical practice may have attenuated the observed treatment effects, particularly under an intention-to-treat framework. These limitations underscore the need for mechanistic and prospective interventional trials to clarify the cardiometabolic and renal effects of GLP-1 RAs in non-obese populations.

## Conclusion

In this BMI-restricted cohort of adults with type 2 diabetes, GLP-1 receptor agonists were associated with lower risks of major kidney events and dialysis initiation, with a generally consistent direction of benefit across clinical subgroups. Cardiovascular outcomes were neutral, but reductions in hospitalization and sepsis suggest additional systemic advantages. These findings indicate that GLP-1 RAs may provide kidney protection even in leaner patients. Randomized trials in low-BMI populations are warranted.

## Supplementary Material

Supplementary methods, figures and tables.

## Figures and Tables

**Table 1:** Baseline characteristics in the population with lower body mass index (<30 Kg/m^2^)

	Before matching			After matching		
Parameter	GLP-1 RA (n = 23,103)	DPP4i (n = 44,456)	SMD	GLP-1 RA (n = 20,928)	DPP4i (n = 20,928)	SMD
BMI (kg/m^2^), mean ± SD	26.6 ± 2.7	25.7 ± 3.3	-	26.6 ± 2.8	26 ± 3.1	-
Age at Index (years), mean ± SD	60.6 ± 11.8	65.1 ± 11.7	0.383	61.4 ± 11.4	61.3 ± 12.2	0.008
Male, n (%)	11,895 (51.5)	24,730 (55.6)	0.083	10,943 (52.3)	11,059 (52.8)	0.011
**Race, *n* (%)**						
White	13,822 (59.8)	22,537 (50.7)	0.184	12,296 (58.8)	12,265 (58.6)	0.003
African American	3,313 (14.3)	6,644 (14.9)	0.017	3,029 (14.5)	3,063 (14.6)	0.005
Asian	1,679 (7.3)	6,194 (13.9)	0.218	1,648 (7.9)	1,589 (7.6)	0.011
Other race	1,539 (6.7)	3,058 (6.9)	0.009	1,394 (6.7)	1,393 (6.7)	< 0.001
**Comorbidity, *n* (%)**						
Hypertensive diseases	13,132 (56.8)	28,399 (63.9)	0.144	12,141 (58)	11,999 (57.3)	0.014
Dyslipidemia	13,467 (58.3)	24,927 (56.1)	0.045	12,079 (57.7)	11,997 (57.3)	0.008
Ischemic heart diseases	3,077 (13.3)	7,860 (17.7)	0.121	2,912 (13.9)	2,877 (13.7)	0.005
Disorders of thyroid gland	3,199 (13.8)	5,595 (12.6)	0.037	2,836 (13.6)	2,765 (13.2)	0.010
Other anxiety disorders	2,057 (8.9)	3,188 (7.2)	0.064	1,796 (8.6)	1,768 (8.4)	0.005
Nicotine dependence	1,549 (6.7)	3,413 (7.7)	0.038	1,444 (6.9)	1,482 (7.1)	0.007
Hepatic failure	1,076 (4.7)	3,466 (7.8)	0.130	1,048 (5)	1,007 (4.8)	0.009
Cerebrovascular diseases	863 (3.7)	2,586 (5.8)	0.098	830 (4)	811 (3.9)	0.005
Fatty liver	603 (2.6)	880 (2)	0.042	515 (2.5)	520 (2.5)	0.002
Gout	391 (1.7)	1,089 (2.5)	0.053	369 (1.8)	369 (1.8)	0.000
Cardiomyopathy	329 (1.4)	931 (2.1)	0.051	313 (1.5)	282 (1.3)	0.013
Pulmonary heart disease	289 (1.3)	997 (2.2)	0.076	284 (1.4)	268 (1.3)	0.007
Atherosclerosis of native arteries of the extremities	288 (1.2)	846 (1.9)	0.053	273 (1.3)	256 (1.2)	0.007
Liver cirrhosis	276 (1.2)	618 (1.4)	0.017	255 (1.2)	267 (1.3)	0.005
Chronic rheumatic heart diseases	223 (1)	787 (1.8)	0.069	220 (1.1)	207 (1)	0.006
**Medication, n (%)**						
Insulin	10,382 (44.9)	16,669 (37.5)	0.152	8,819 (42.1)	8,724 (41.7)	0.009
Biguanides	12919 (58.8)	26,643 (64.9)	0.126	1,1596 (60.7)	11,692 (61.2)	0.010
Sulfonylureas	5,191 (22.5)	13,695 (30.8)	0.189	4,975 (23.8)	4,931 (23.6)	0.005
SGLT2i	6,114 (26.5)	6,485 (14.6)	0.297	4,867 (23.3)	4,813 (23)	0.006
Thiazolidinediones	1,392 (6)	2,490 (5.6)	0.018	1,219 (5.8)	1,223 (5.8)	0.001
Statin	14,091 (61)	28,915 (65)	0.084	12,818 (61.2)	12,786 (61.1)	0.003
Fibrates	1,312 (5.7)	2,468 (5.6)	0.006	1,193 (5.7)	1,161 (5.5)	0.007
RASB	11,810 (51.1)	24,922 (56.1)	0.099	10,837 (51.8)	10,749 (51.4)	0.008
Beta-blocker	6,280 (27.2)	16,576 (37.3)	0.217	6,009 (28.7)	5,878 (28.1)	0.014
Calcium channel blockers	4,374 (18.9)	12,005 (27)	0.193	4,207 (20.1)	4,110 (19.6)	0.012
Antithrombotic agents	7,920 (34.3)	20,963 (47.2)	0.264	7,574 (36.2)	7,477 (35.7)	0.010
NSAID	4,908 (21.2)	9,468 (21.3)	0.001	4,413 (21.1)	4,412 (21.1)	< 0.001
Immunosuppressants	577 (2.5)	1,051 (2.4)	0.009	504 (2.4)	499 (2.4)	0.002
Immunostimulants	15 (0.1)	51 (0.1)	0.017	15 (0.1)	14 (0.1)	0.002
**Laboratory, mean ± SD**						
eGFR (mL/min/1.73m2)	78.4 ± 29.8	73.4 ± 32.5	0.161	77.5 ± 29.8	79.4 ± 31.8	0.060
Urea nitrogen (mg/dL)	18.4 ± 9.9	20.4 ± 12.8	0.176	18.6 ± 10.1	18.3 ± 10.7	0.025
Sodium (mmol/L)	138 ± 3.3	137.9 ± 3.7	0.015	138 ± 3.3	137.9 ± 3.5	0.017
Potassium (mmol/L)	4.3 ± 0.5	4.3 ± 0.5	0.010	4.3 ± 0.5	4.3 ± 0.5	0.005
Calcium (mg/dL)	9.4 ± 0.6	9.3 ± 0.7	0.203	9.4 ± 0.6	9.4 ± 0.6	0.034
Phosphate (mg/dL)	3.4 ± 0.9	3.5 ± 1	0.009	3.4 ± 0.9	3.4 ± 1	0.009
Albumin (g/dL)	4.1 ± 0.6	4 ± 0.6	0.248	4.1 ± 0.6	4.1 ± 0.6	0.014
Hemoglobin (g/dL)	13.5 ± 2.1	12.7 ± 2.3	0.348	13.4 ± 2.1	13.4 ± 2.1	0.023
Hemoglobin A1c (%)	8.7 ± 2.3	8.3 ± 2.1	0.203	8.6 ± 2.3	8.6 ± 2.2	0.010
ALT (U/L)	28.6 ± 27.8	29.1 ± 59.8	0.010	28.6 ± 28.2	29.1 ± 50.7	0.013
AST (U/L)	24.9 ± 24.8	27.2 ± 69	0.043	25 ± 25.4	25.7 ± 52.6	0.017
Alkaline phosphatase (U/L)	87.9 ± 45	86.7 ± 51.2	0.024	88 ± 45.9	85.8 ± 48.5	0.045
LDL (mg/dL)	94.6 ± 42.3	91.3 ± 40.2	0.078	94.1 ± 42.4	94.3 ± 40.8	0.004
HDL (mg/dL)	45.5 ± 15.7	44.7 ± 16.6	0.052	45.6 ± 15.8	44.4 ± 16.4	0.076
Triglyceride (mg/dL)	188.2 ± 249.5	170.6 ± 173.9	0.082	183.9 ± 221.5	181.8 ± 198.3	0.010
C reactive protein (mg/L)	41.5 ± 71.9	46.7 ± 69.2	0.074	43.7 ± 73	41.3 ± 67.1	0.034
UACR (mg/g)	639.4 ± 13881.5	1187.4 ± 36154	0.020	702.3 ± 14741.3	310 ± 3253.7	0.037

Abbreviation: BMI, body mass index; GLP-1 RA, glucagon-like peptide-1 receptor agonists; DPP4i, dipeptidyl peptidase-4 inhibitor; SGLT2i, sodium-glucose co-transporter 2 Inhibitors; RASB, renin-angiotensin system blockade; NSAID, nonsteroidal anti-inflammatory drug.; eGFR, estimated glomerular filtration rate; ALT, alanine aminotransferase; AST, aspartate aminotransferase; LDL, low density lipoprotein; HDL, high density lipoprotein; UACR, urine albumin / creatinine ratio;

**Table 2:** Clinical outcomes in type 2 diabetes patients with low BMI (<30 kg/m^2^)

Clinical Outcomes	GLP-1 RA user (n = 20,928)		DPP4i user (n = 20,928)		GLP-1 RA vs. DPP4i			
	Events (n)	%	Events (n)	%	HR (95%CI)	*P value*	FDR-corrected p value	E-value
**Primary outcome**								
^#^MAKE	3,097	14.8	3,507	16.8	0.93 (0.89-0.98)	0.005^†^	0.011	1.36
AKI	2,218	10.6	2,446	11.7	0.97 (0.91-1.02)	0.222^†^	0.285	1.21
ESKD on dialysis	573	2.7	765	3.7	0.78 (0.7-0.87)	< 0.001^†^	0.009	1.88
Mortality	987	4.7	1,069	5.1	1.01 (0.93-1.1)	0.818	0.888	1.11
**Secondary outcome**								
AMI	862	4.1	863	4.1	1.08 (0.98-1.18)	0.121	0.182	1.37
Heart failure	2,125	10.2	2,252	10.8	0.99 (0.94-1.05)	0.789	0.888	1.11
Stroke	950	4.5	944	4.5	1.08 (0.99-1.18)	0.086	0.155	1.37
Hospitalization	4,358	20.8	5,347	25.5	0.84 (0.8-0.87)	< 0.001^†^	0.009	1.67
Sepsis	1,149	5.5	1,401	6.7	0.88 (0.81-0.95)	0.001	0.009	1.53

#MAKE includes acute kidney injury, end stage of kidney disease, entering dialysis, and death. †This indicate the proportional hazard assumption is violated. Abbreviation: HR, hazard ratio; CI, confidence interval; MAKE, major adverse kidney events; AKI, acute kidney injury; ESKD, end stage of kidney disease; AMI, acute myocardial infarction; FDR, Benjamini-Hochberg false discovery rate.

**Table 3:** Clinical outcomes analysis by different BMI level groups

Clinical Outcomes	BMI 25-30 kg/m^2^			BMI <25 kg/m^2^		
	GLP-1 RA vs. DPP4i			GLP-1 RA vs. DPP4i (n = 2,482 parings)		
	HR (95%CI)	*P value*	E-value	HR (95%CI)	*P value*	E-value
**Primary outcome**						
^#^MAKE	0.92 (0.86-0.99)	0.022	1.39	0.91 (0.80-1.04)	0.163	1.43
AKI	0.92 (0.84-0.99)	0.030	1.39	0.96 (0.82-1.13)	0.627	1.25
ESKD on dialysis	0.87 (0.74-1.01)	0.074	1.56	0.72 (0.54-0.95)	0.021	2.12
Mortality	0.99 (0.87-1.12)	0.814	1.11	1.04 (0.82-1.33)	0.742	1.24
**Secondary outcome**						
AMI	0.98 (0.86-1.13)	0.817	1.16	1.02 (0.76-1.36)	0.917	1.16
Heart failure	1.01 (0.93-1.10)	0.747	1.11	1.04 (0.88-1.24)	0.649	1.24
Stroke	0.98 (0.86-1.12)	0.806	1.16	1.13 (0.87-1.47)	0.351	1.51
Hospitalization	0.84 (0.79-0.86)	<0.001	1.67	0.81 (0.73-0.90)	<0.001	1.77
Sepsis	0.81 (0.72-0.90)	<0.001	1.77	0.80 (0.65-0.98)	0.027	1.81

#MAKE includes acute kidney injury, end stage of kidney disease, entering dialysis, and death. †This indicate the proportional hazard assumption is violated. Abbreviation: HR, hazard ratio; CI, confidence interval; MAKE, major adverse kidney events; AKI, acute kidney injury; ESKD, end stage of kidney disease; AMI, acute myocardial infarction.

## Data Availability

The data utilized in this study were obtained from the TriNetX Global Health Research Network and are not publicly available due to licensing agreements and privacy regulations. TriNetX provides access to de-identified, aggregate-level data sourced from a global consortium of healthcare institutions. Researchers interested in accessing the data may submit a request via the TriNetX website (https://trinetx.com) or by contacting Privacy@TriNetX.com. Additionally, data may be made available from the corresponding author upon reasonable request.
