# Supplementary material for: Clinical effects of glucagon-like peptide-1 receptor agonist in type 2 diabetes with low body mass index: findings from large-scale emulated target trials
Source: Int J Med Sci. 2026 Mar 30;23(5):1748–60. doi: 10.7150/ijms.130295 (PMC13133889; doi:10.7150/ijms.130295)
Supplement: Supplementary file 1 — Supplementary methods, figures and tables. [file ijmsv23p1748s1.pdf]

Supplementary materials

Method S1. Codes for cohorts, outcomes, and baseline data..2

Table S1. Target trial emulation..12

Table S2. Different models for propensity score matching 13

Table S3. Different time frames on the clinical outcome analysis..............................15

Table S4. Clinical outcomes analysis with a 3-month lag after index day 17

Table S5. Sensitivity analysis including patients with cardiovascular disease within 6 months prior to the indexdate……………………………………………………19

Figure S1. Cohort Construction and Index-Date Definitions. 21

Figure S2. Kaplan–Meier curves for kidney and mortality 22

Figure S4. Subgroup Analyses for MAKE 24

Figure S5. Subgroup Analyses for ESKD on Dialysis...25

Figure S6. Distribution of Follow-up Time After Matching. 26

Method S1. Codes for cohorts, outcomes, and baseline data

1.Codes for cohorts

This section lists all terms used in the definitions of the two cohorts.

1.1 Query Criteria for Cohort 1 (query name: GLP-1 RA with lower BMI)

**Group 1**				
**visit>2**				
must have	visit	TNX:Visit	Visit	
number of instances	Greater than or equal to 2 instances			
date constraint	The terms in this group occurred at any time			
**Group 2**				
**T2DM with age >18 years**				
must have	diagnosis	UMLS:ICD10CM:E11	Type 2 diabetes mellitus (at least 18 years old at event)	
number of instances	Greater than or equal to 2 instances			
date constraint	The terms in this group occurred between Jan 1, 2016 and Dec 31, 2023			
**Group 3**				
**Lower BMI**				
must have	laboratory	TNX:9083	BMI (at most 30.00 kg/m2 (most recent occurrence))	
date constraint	The terms in this group occurred between Jan 1, 2016 and Dec 31, 2023			
**Group 4**				
**Group 4A Lower BMI**				
must have	laboratory	TNX:9083	BMI (at most 30.00 kg/m2)	
date constraint	The terms in this group occurred between Jan 1, 2016 and Dec 31, 2023			
event relationship	Any instance of Group 4B occurred within 6 months on or after the first instance of Lower BMI			

**Group 4B GLP-1 RA use**				
must have		medication	NLM:ATC:A10BJ	Glucagon-like peptide-1 (GLP-1) analogues
**Group 5**				
Group 5A **:GLP-1 RA use**				
must have		medication	NLM:ATC:A10BJ	Glucagon-like peptide-1 (GLP-1) analogues
date constraint		The terms in this group occurred between Jan 1, 2016 and Dec 31, 2023		
event relationship		Any instance of Group 5B occurred on the same date as the first instance of Group 5A		
**Group 5B No DPP-4i use at the same day**				
cannot have		medication	NLM:ATC:A10BH	Dipeptidyl peptidase 4 (DPP-4) inhibitors
**Group 6**				
Group 6A: **GLP-1 RA use**				
must have		medication	NLM:ATC:A10BJ	Glucagon-like peptide-1 (GLP-1) analogues
date constraint		The terms in this group occurred between Jan 1, 2016 and Dec 31, 2023		
event relationship		Any instance of Group 6B occurred within 1 year and 1 day before the first instance of Group 6A		
**Group 6B: no GLP-1 RA and DPP-4i use one year before the index day**				
cannot have		medication	NLM:ATC:A10BJ	Glucagon-like peptide-1 (GLP-1) analogues
	or	medication	NLM:ATC:A10BH	Dipeptidyl peptidase 4 (DPP-4) inhibitors
**Group 7**				
**Group 7A GLP-1 RA use**				
must have		medication	NLM:ATC:A10BJ	Glucagon-like peptide-1 (GLP-1) analogues
date constraint		The terms in this group occurred between Jan 1, 2016 and Dec 31, 2023		
event relationship		Any instance of Group 7B occurred within 1 year on or before the first instance of Group 7A		
**Group 7B: exclude dialysis**				
cannot have		procedure	UMLS:CPT:90945	Dialysis procedure other than hemodialysis (eg, peritoneal

				dialysis, hemofiltration, or other continuous renal replacement therapies), with single evaluation by a physician or other qualified health care professional
	or	procedure	UMLS:CPT:1012740	Dialysis Services and Procedures
**Group 8**				
**Group 8A GLP-1 RA use**				
must have		medication	NLM:ATC:A10BJ	Glucagon-like peptide-1 (GLP-1) analogues
date constraint		The terms in this group occurred between Jan 1, 2016 and Dec 31, 2023		
event relationship		Any instance of Group 8B occurred on or before the first instance of Group 8A		
**Group 8B exclude transplants**				
cannot have		diagnosis	UMLS:ICD10CM:Z94	Transplanted organ and tissue status
**Group 9**				
**Group 9A GLP-1 RA use**				
must have		medication	NLM:ATC:A10BJ	Glucagon-like peptide-1 (GLP-1) analogues
date constraint		The terms in this group occurred between Jan 1, 2016 and Dec 31, 2023		
event relationship		Any instance of Group 9B occurred on or before the first instance of Group 9A		
**Group 9B exclude any neoplasms**				
cannot have		diagnosis	UMLS:ICD10CM:C00-D49	Neoplasms
**Group 10**				
**Group 10A GLP-1 RA use**				
must have		medication	NLM:ATC:A10BJ	Glucagon-like peptide-1 (GLP-1) analogues
date constraint		The terms in this group occurred between Jan 1, 2016 and Dec 31, 2023		
event relationship		Any instance of Group 10B occurred within 6 months on or before the first instance of Group 10A		

Group 10B exclude CVD within 6 months before index day				
cannot have		diagnosis	UMLS:ICD10CM:I21	Acute myocardial infarction
	or	diagnosis	UMLS:ICD10CM:I20.0	Unstable angina
	or	diagnosis	UMLS:ICD10CM:I63	Cerebral infarction
	or	diagnosis	UMLS:ICD10CM:G45.9	Transient cerebral ischemic attack, unspecified

1.2 Query Criteria for Cohort 2 (DPP-4i with lower BMI)

Group 1				
visit>2				
must have		visit	TNX:Visit	Visit
number of instances		Greater than or equal to 2 instances		
date constraint		The terms in this group occurred at any time		
**Group 2**				
**T2DM with age >18 years**				
must have		diagnosis	UMLS:ICD10CM:E11	Type 2 diabetes mellitus (at least 18 years old at event)
number of instances		Greater than or equal to 2 instances		
date constraint		The terms in this group occurred between Jan 1, 2016 and Dec 31, 2023		
**Group 3**				
**Lower BMI**				
must have		laboratory	TNX:9083	BMI (at most 30.00 kg/m2 (most recent occurrence))
date constraint		The terms in this group occurred between Jan 1, 2016 and Dec 31, 2023		
**Group 4**				
**Group 4A Lower BMI**				
must have		laboratory	TNX:9083	BMI (at most 30.00 kg/m2)
date constraint		The terms in this group occurred between Jan 1, 2016 and Dec 31, 2023		
event relationship		Any instance of Group 4B occurred within 6 months on or after the		

	first instance of Advance CKD			
**Group 4B DPP4i use**				
must have		medication	NLM:ATC:A10BH	Dipeptidyl peptidase 4 (DPP-4) inhibitors
**Group 5**				
**Group 5A DPP4i use**				
must have		medication	NLM:ATC:A10BH	Dipeptidyl peptidase 4 (DPP-4) inhibitors
date constraint	The terms in this group occurred between Jan 1, 2016 and Dec 31, 2023			
event relationship	Any instance of Group 5B occurred on the same date as the first instance of Group 5A			
**Group 5B no GLP-1 RA use at the same day**				
cannot have		medication	NLM:ATC:A10BJ	Glucagon-like peptide-1 (GLP-1) analogues
**Group 6**				
**Group 6A DPP4i use**				
must have		medication	NLM:ATC:A10BH	Dipeptidyl peptidase 4 (DPP-4) inhibitors
date constraint	The terms in this group occurred between Jan 1, 2016 and Dec 31, 2023			
event relationship	Any instance of Group 6B occurred within 1 year and 1 day before the first instance of Group 6A			
**Group 6B no GLP-1 RA and DPP-4i use one year before the index day**				
cannot have		medication	NLM:ATC:A10BJ	Glucagon-like peptide-1 (GLP-1) analogues
	or	medication	NLM:ATC:A10BH	Dipeptidyl peptidase 4 (DPP-4) inhibitors
**Group 7**				
**Group 7A DPP4i use**				
must have		medication	NLM:ATC:A10BH	Dipeptidyl peptidase 4 (DPP-4) inhibitors
date constraint	The terms in this group occurred between Jan 1, 2016 and Dec 31, 2023			
event relationship	Any instance of Group 7B occurred within 1 year on or before the first instance of Group 7A			
**Group 7B exclude dialysis**				
cannot		procedure	UMLS:CPT:90945	Dialysis procedure other than

have				hemodialysis (eg, peritoneal dialysis, hemofiltration, or other continuous renal replacement therapies), with single evaluation by a physician or other qualified health care professional
	or	procedure	UMLS:CPT:1012740	Dialysis Services and Procedures
**Group 8**				
**Group 8A DPP4i use**				
must have		medication	NLM:ATC:A10BH	Dipeptidyl peptidase 4 (DPP-4) inhibitors
date constraint		The terms in this group occurred between Jan 1, 2016 and Dec 31, 2023		
event relationship		Any instance of Group 8B occurred on or before the first instance of Group 8A		
**Group 8B exclude transplants**				
cannot have		diagnosis	UMLS:ICD10CM:Z94	Transplanted organ and tissue status
**Group 9**				
**Group 9A DPP4i use**				
must have		medication	NLM:ATC:A10BH	Dipeptidyl peptidase 4 (DPP-4) inhibitors
date constraint		The terms in this group occurred between Jan 1, 2016 and Dec 31, 2023		
event relationship		Any instance of Group 9B occurred on or before the first instance of Group 9A		
**Group 9B exclude any neoplasms**				
cannot have		diagnosis	UMLS:ICD10CM:C00-D49	Neoplasms
**Group 10**				
**Group 10A DPP4i use**				
must have		medication	NLM:ATC:A10BH	Dipeptidyl peptidase 4 (DPP-4) inhibitors
date constraint		The terms in this group occurred between Jan 1, 2016 and Dec 31, 2023		
event relationship		Any instance of Group 10B occurred within 6 months on or before		

		the first instance of Group 10A		
**Group 10B**	**exclude CVD within 6 months before index day**			
cannot have		diagnosis	UMLS:ICD10CM:I21	Acute myocardial infarction
	or	diagnosis	UMLS:ICD10CM:I63	Cerebral infarction
	or	diagnosis	UMLS:ICD10CM:G45.9	Transient cerebral ischemic attack, unspecified
	or	diagnosis	UMLS:ICD10CM:I20.0	Unstable angina

2. Codes for outcome definitions

Table below outlines the definitions for each outcome and the analysis specifications. For outcome definitions consisting of more than one term, at least one term must match.

**MACE**		
Diagnosis	UMLS:ICD10CM:I21	Acute myocardial infarction
Diagnosis	UMLS:ICD10CM:I50	Heart failure
Demographics	Deceased	Deceased
Diagnosis	UMLS:ICD10CM:I63	Cerebral infarction
Diagnosis	UMLS:ICD10CM:I62	Other and unspecified nontraumatic intracranial hemorrhage
Diagnosis	UMLS:ICD10CM:I46	Cardiac arrest
**AMI**		
Diagnosis	UMLS:ICD10CM:I21	Acute myocardial infarction
**Heart failure**		
Diagnosis	UMLS:ICD10CM:I50	Heart failure
**Stroke**		
Diagnosis	UMLS:ICD10CM:I63	Cerebral infarction
Diagnosis	UMLS:ICD10CM:I62	Other and unspecified nontraumatic intracranial hemorrhage
**Mortality**		
Demographics	Deceased	Deceased

**sepsis**		
Diagnosis	UMLS:ICD10CM:A40	Streptococcal sepsis
Diagnosis	UMLS:ICD10CM:A41	Other sepsis
Diagnosis	UMLS:ICD10CM:R65.20	Severe sepsis without septic shock
Diagnosis	UMLS:ICD10CM:A20.7	Septicemic plague
Diagnosis	UMLS:ICD10CM:R65.21	Severe sepsis with septic shock
Diagnosis	UMLS:ICD10CM:R78.81	Bacteremia
**Hospitalization**		
Visit	UMLS:HL7V3.0:VisitType:IMP	Visit: Inpatient Encounter
Visit	UMLS:HL7V3.0:VisitType:NONAC	Visit: Inpatient Non-acute
**MAKE**		
Diagnosis	UMLS:ICD10CM:N18.6	End stage renal disease
Diagnosis	UMLS:ICD10CM:N17	Acute kidney failure
Procedure	UMLS:CPT:1012740	Dialysis Services and Procedures
Procedure	UMLS:CPT:90945	Dialysis procedure other than hemodialysis (eg, peritoneal dialysis, hemofiltration, or other continuous renal replacement therapies), with single evaluation by a physician or other qualified health care professional
Demographics	Deceased	Deceased
**ESRD on dialysis**		
Diagnosis	UMLS:ICD10CM:N18.6	End stage renal disease
Diagnosis	UMLS:ICD10CM:Z99.2	Dependence on renal dialysis
**Acute kidney injury**		
Diagnosis	UMLS:ICD10CM:N17	Acute kidney failure

3. codes for the baseline covariates for propensity score matching

**Demographics**	
AI	Age at Index
2106-3	White people
2054-5	African American
2028-9	Asian

2131-1	Other Race
M	Male
**Diagnosis**	
International Classification of Diseases, Tenth Revision,	Name
E78	Disorders of lipoprotein metabolism and other lipidemias
I50	Heart failure
I10-I1A	Hypertensive diseases
I42	Cardiomyopathy
I20-I25	Ischemic heart diseases
I60-I69	Cerebrovascular diseases
I26-I28	Pulmonary heart disease and diseases of pulmonary circulation
I05-I09	Chronic rheumatic heart diseases
E00-E07	Disorders of thyroid gland
K74	Fibrosis and cirrhosis of liver
F17	Nicotine dependence
M10	Gout
F41	Other anxiety disorders
K76.0	Fatty (change of) liver, not elsewhere classified
I70.2	Atherosclerosis of native arteries of the extremities
**Medication**	
Anatomical Therapeutic Chemical	Name
A10A	INSULINS AND ANALOGUES
A10BB	Sulfonylureas
A10BG	Thiazolidinediones
C10AA	HMG CoA reductase inhibitors
C10AB	Fibrates
C09	AGENTS ACTING ON THE RENIN-ANGIOTENSIN SYSTEM
C07	BETA BLOCKING AGENTS

C08	CALCIUM CHANNEL BLOCKERS
A10BK	Sodium-glucose co-transporter 2 (SGLT2) inhibitors
M01A	ANTIINFLAMMATORY AND ANTIRHEUMATIC PRODUCTS, NON-STEROIDS
B01	ANTITHROMBOTIC AGENTS
L03	IMMUNOSTIMULANTS
L04	IMMUNOSUPPRESSANTS

Laboratory data

TNX, Curated	Name (unit)	Data missing rate after matching with full baseline covariates	
		GLP- RAs	DPP-4is
9029	Sodium in Serum (mmol/L)	46.6%	42.8%
9028	Potassium in Serum (mmol/L)	45%	41.7%
9030	Urea nitrogen (mg/dL)	49.1%	44.6%
9022	Calcium in Serum (mg/dL)	47.4%	43.8%
9027	Phosphate in Serum (mg/dL)	92%	87.4%
9014	Hemoglobin in Blood (g/dL)	59.2%	52.3%
9044	Alanine aminotransferase in Serum (U/L)	51.8%	50.1%
9047	Aspartate aminotransferase in Serum (U/L)	52.8%	50.4%
9046	Alkaline phosphatase in Serum (U/L)	54.3%	51.9%
9045	Albumin in Serum (g/dL)	54.6%	52.5%
9002	Cholesterol in LDL in Serum (mg/dL)	64.2%	67.6%
9001	Cholesterol in HDL in Serum (mg/dL)	62.8%	66.7%
9004	Triglyceride in Serum (mg/dL)	63.3%	66.7%
9037	Hemoglobin A1c/Hemoglobin.total in Blood (%)	50.3%	54.6%
9083	BMI (kg/m^2)	0.1%	0.1%
9063	C reactive protein in Serum (mg/L)	95.9%	94.9%
8001	Glomerular filtration rate/1.73 sq M.predicted in Serum, by Creatinine-based formula (MDRD) (mL·min^{-1}·1.73 m^{-2})	45.1%	41.3%
9318-7	Albumin/Creatinine in Urine (mg/g creat)	95.1%	96.5%

Table S1. Target trial emulation

Approach	Target Trial	Target Trial Emulation
**Eligibility criteria**	Adults with type 2 diabetes and BMI ≤ 30 kg/m², no prior GLP-1 RA or DPP-4i exposure within 1 year before randomization; excluded if history of dialysis, transplant, malignancy, recent CVD, or concurrent comparator drug use.	Adults aged ≥ 18 years with type 2 diabetes and BMI ≤ 30 kg/m², at least 2 clinical visits (2016–2023) in TriNetX; excluded if prior GLP-1 RA/DPP-4i use in prior year, dialysis, transplant, malignancy, recent CVD, or concurrent comparator use.
**Treatment strategies**	Initiation of GLP-1 RA therapy versus DPP-4 inhibitor therapy.	New users of GLP-1 RA (n = 23,103) vs DPP-4i (n = 44,156); post-matching: 20,928 each, 1:1 propensity-score-matched.
**Treatment assignment**	Random assignment to GLP-1 RA or DPP-4i.	Observed treatment initiation in real-world data; assignment not randomized but balanced using PSM across baseline covariates.
**Outcomes**	Primary: Major Adverse Kidney Events (MAKE: acute kidney injury, end-stage kidney disease, dialysis, or death). Secondary: individual renal, cardiovascular (AMI, stroke, HF), mortality, hospitalization, sepsis.	Primary and secondary outcomes measured from EHR data via TriNetX with ICD codes; composite and isolated kidney and cardiovascular outcomes derived as listed.
**Follow-up**	From treatment initiation until outcome, loss to follow-up, or 4 years.	Index date = first prescription; outcomes tracked 1 day – 4 years post-index (through Aug 17, 2025).
**Causal contrasts**	Intention-to-treat effect (GLP-1 RA vs DPP-4i).	New-user, active-comparator, intention-to-treat design; effect estimated within matched cohorts.
**Statistical analysis**	Compare hazard ratios for MAKE and secondary outcomes between treatment arms using Cox proportional hazards regression and Kaplan–Meier curves.	Hazard ratios from Cox regression, proportionality confirmed via Schoenfeld test; Kaplan–Meier event-free curves plotted. Sensitivity analyses: alternate PSM models, time-window checks, latency (3 month lags).

Table S2. Different models for propensity score matching

Clinical Outcomes	After propensity score matching with 4-year follow-up					
	Model 1		Model 2		Model 3	
	HR (95%CI)	*P value*	HR (95%CI)	*P value*	HR (95%CI)	*P value*
**Primary outcome**						
#MAKE	0.8 (0.76–0.83)	<0.001†	0.9 (0.86–0.94)	<0.001†	0.89 (0.85–0.93)	<0.001†
**Secondary outcome**						
Entering dialysis	0.57 (0.51–0.63)	<0.001†	0.67 (0.61–0.74)	<0.001†	0.73 (0.65–0.81)	<0.001†
Acute kidney injury	0.83 (0.78–0.87)	<0.001†	0.94 (0.89–1.00)	0.038 †	0.91 (0.86–0.97)	0.001†
Mortality	0.91 (0.83–0.98)	0.017	0.98 (0.90–1.06)	0.556	0.97 (0.89–1.05)	0.420†
AMI	0.95 (0.87–1.03)	0.223	1.02 (0.93–1.12)	0.648	1.06 (0.97–1.16)	0.209
Heart failure	0.86 (0.82–0.91)	<0.001†	1.02 (0.97–1.09)	0.425	1.01 (0.95–1.07)	0.834
Stroke	0.97 (0.89–1.06)	0.521	1.09 (1.00–1.19)	0.056	1.05 (0.96–1.15)	0.257
Hospitalization	0.73 (0.71–0.76)	<0.001†	0.79 (0.76–0.82)	<0.001†	0.79 (0.76–0.82)	<0.001†
Sepsis	0.77 (0.72–0.83)	<0.001†	0.85 (0.79–0.92)	<0.001†	0.82 (0.76–0.89)	<0.001†

Model 1 adjusts for age, sex, race, and body mass index in propensity score matching. Model 2 builds on Model 1 by adding baseline

comorbidities, while Model 3 further includes baseline medication use.

#MAKE includes acute kidney injury, end stage of kidney disease, entering dialysis, and death.

†This indicate the proportional hazard assumption is violated.

Abbreviation: HR, hazard ratio; CI, confidence interval; MAKE, major adverse kidney events; AMI, acute myocardial infarction.

Table S3. Different time frames on the clinical outcome analysis

Clinical Outcomes	After propensity score matching					
	1 day to 1 years		1 day to 2 years		1 day to 3 years	
	HR (95%CI)	*P value*	HR (95%CI)	*P value*	HR (95%CI)	*P value*
**Primary outcome**						
#MAKE	0.83 (0.77–0.89)	<0.001†	0.88 (0.83–0.93)	<0.001†	0.91 (0.86–0.96)	<0.001†
**Secondary outcome**						
Entering dialysis	0.62 (0.53–0.72)	<0.001†	0.69 (0.61–0.78)	<0.001†	0.75 (0.67–0.85)	<0.001†
Acute kidney injury	0.9 (0.83–0.99)	0.021	0.94 (0.87–1.01)	0.080†	0.96 (0.90–1.02)	0.194
Mortality	0.91 (0.77–1.06)	0.213	0.88 (0.79–0.99)	0.039	0.92 (0.83–1.02)	0.118
AMI	0.94 (0.81–1.10)	0.426	0.92 (0.81–1.04)	0.172	0.98 (0.87–1.09)	0.667
Heart failure	0.92 (0.84–0.99)	0.036	0.95 (0.88–1.02)	0.123	0.97 (0.91–1.03)	0.317
Stroke	0.97 (0.83–1.12)	0.650	0.95 (0.84–1.07)	0.381	0.99 (0.89–1.10)	0.858
Hospitalization	0.77 (0.73–0.81)	<0.001	0.78 (0.74–0.81)	<0.001	0.79 (0.75–0.82)	<0.001†
Sepsis	0.82 (0.73–0.93)	0.002	0.86 (0.78–0.94)	0.002	0.88 (0.81–0.96)	0.004

[†]This indicate the proportional hazard assumption is violated.

[#]MAKE includes acute kidney injury, end stage of kidney disease, entering dialysis, and death

Abbreviation: HR, hazard ratio; CI, confidence interval; MAKE, major adverse kidney events; AMI, acute myocardial infarction

Table S4. Clinical outcomes analysis with a 3-month lag after index day

Clinical Outcomes	GLP-1 RA user (n = 20,928)		DPP4 user (n = 20,928)		GLP-1 RA vs. DPP-4i	
	Events (n)	%	Events (n)	%	HR (95%CI)	*P value*
**Primary outcome**						
#MAKE	2,831	14.8	3,166	16.6	0.95 (0.90–1.00)	0.045
**Secondary outcome**						
Entering dialysis	533	2.8	678	3.5	0.82 (0.74–0.92)	0.001†
Acute kidney injury	2,031	10.6	2,214	11.6	0.98 (0.92–1.04)	0.505
Mortality	906	4.7	958	5	1.04 (0.95–1.14)	0.382
AMI	728	3.8	752	3.9	1.06 (0.96–1.17)	0.281
Heart failure	1,927	10.1	2,027	10.6	1.00 (0.94–1.07)	0.980
Stroke	782	4.1	813	4.3	1.04 (0.94–1.15)	0.463
Hospitalization	4,291	22.4	5,277	27.6	0.83 (0.80–0.87)	<0.001†
Sepsis	1,040	5.4	1,286	6.7	0.87 (0.80–0.94)	0.001

#MAKE includes acute kidney injury, end stage of kidney disease, entering dialysis, and death.

† This indicate the proportional hazard assumption is violated.

Abbreviation: HR, hazard ratio ; CI, confidence interval; MAKE, major adverse kidney events; AMI, acute myocardial infarction.

Table S5. Sensitivity analysis including patients with cardiovascular disease within 6 months prior to the index date.

Clinical Outcomes	Type 2 diabetes patients with low BMI (<30 kg/m^2) after matching							
	GLP-1 RA user (n = 20,928)		DPP4i user (n = 20,928)		GLP-1 RA vs. DPP4i			
	Events (n)	%	Events (n)	%	HR (95%CI)	*P value*	FDR-corrected p value	E-value
**Primary outcome**								
#MAKE	3,143	15.9	3,524	17.8	0.93 (0.88–0.97)	0.002	0.006	1.38
Acute kidney injury	2,317	11.7	2,500	12.6	0.97 (0.92–1.02)	0.527	0.592	1.23
ESKD on dialysis	615	3.1	743	3.8	0.86 (0.77–0.95)	0.005	0.011	1.6
Mortality	994	5.0	1,180	5.6	0.90 (0.83–0.98)	0.015	0.027	1.47
**Secondary outcome**								
AMI	1,005	5.1	1,129	5.7	0.93 (0.85–1.01)	0.096	0.144	1.38
Heart failure	2,166	10.9	2,264	11.4	0.99 (0.93–1.05)	0.741	0.741	1.12
Stroke	1,315	6.6	1,321	6.7	1.04 (0.96–1.12)	0.357	0.459	1.26
Hospitalization	4,666	23.6	5,596	28.3	0.84 (0.81–0.88)	<0.001	0.004	1.67
Sepsis	1,166	5.9	1,431	7.2	0.85 (0.79–0.92)	<0.001†	0.004	1.63

[#]MAKE includes acute kidney injury, end stage of kidney disease, entering dialysis, and death.

[†]This indicate the proportional hazard assumption is violated.

Abbreviation: HR, hazard ratio; CI, confidence interval; MAKE, major adverse kidney events; AMI, acute myocardial infarction; FDR, Benjamini-Hochberg false discovery rate

Figure S1. Cohort Construction and Index-Date Definitions**.**

Abbreviations: GLP-1 RA, glucagon-like peptide-1 receptor agonist; DPP-4i, dipeptidyl peptidase-4 inhibitor; Rx, prescription.

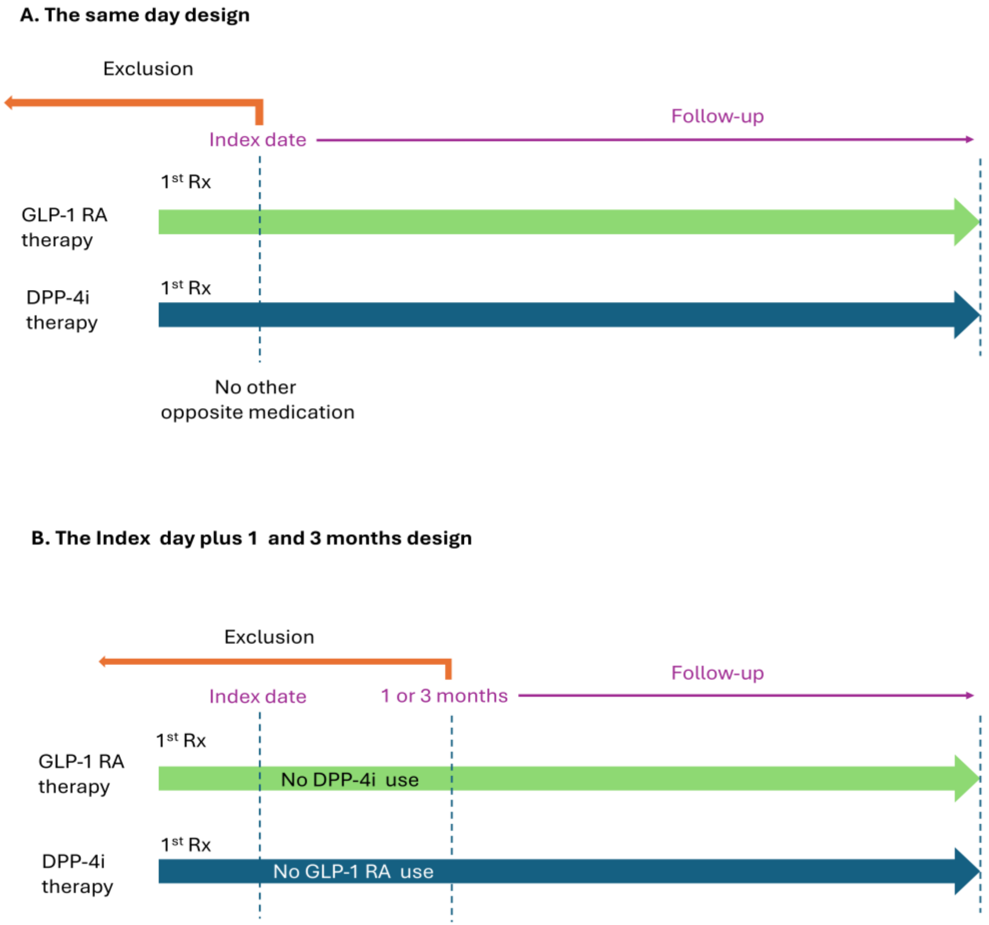

Figure S2. Kaplan–Meier curves for kidney and mortality .

Abbreviations: GLP-1 RA, glucagon-like peptide-1 receptor agonist; DPP-4i, dipeptidyl peptidase-4 inhibitor; AKI, acute kidney injury

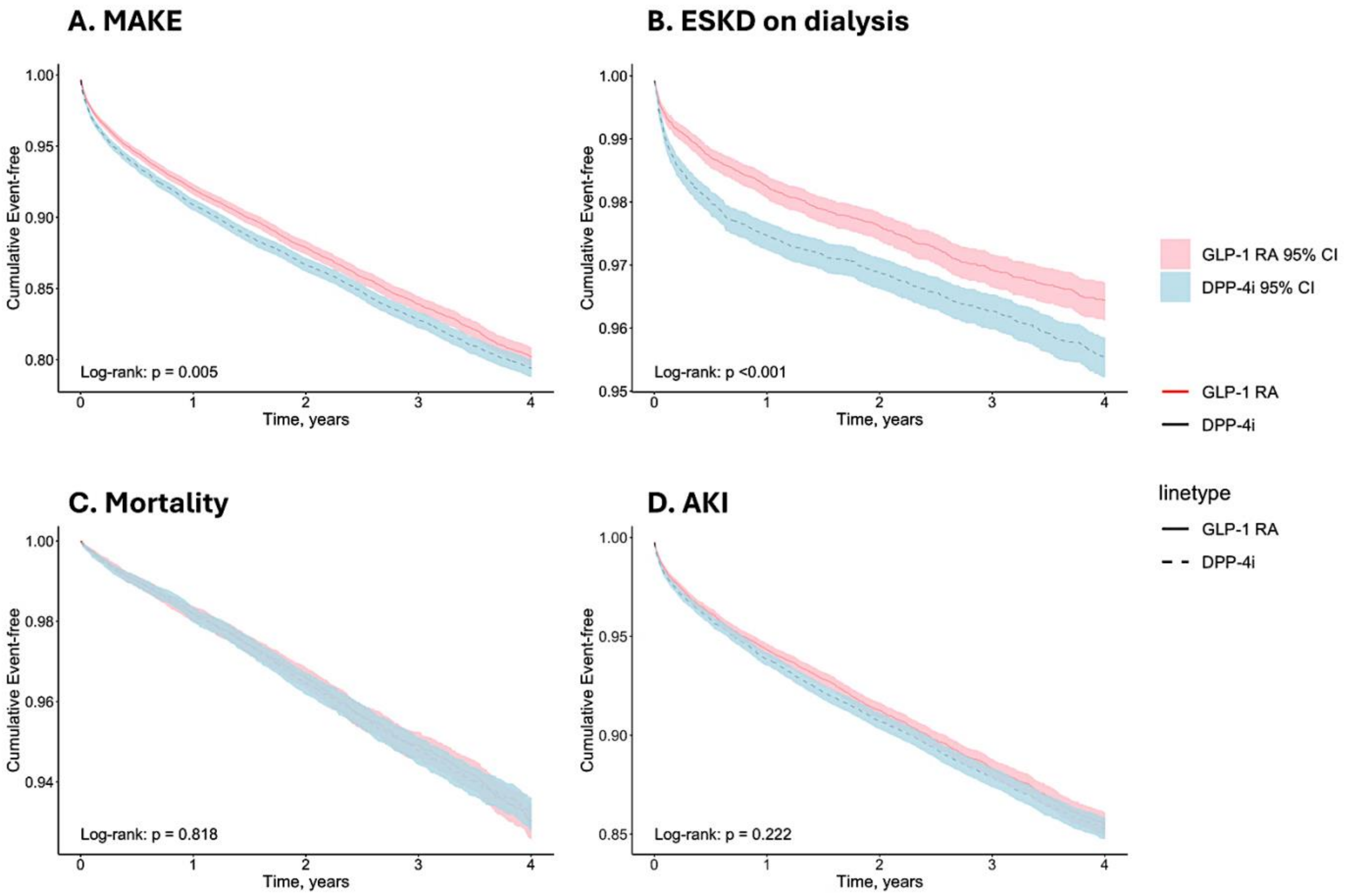

Figure S3. Kaplan–Meier Curves for Cardiovascular, Hospitalization, and Sepsis Outcomes

Abbreviations: GLP-1 RA, glucagon-like peptide-1 receptor agonist; DPP-4i, dipeptidyl peptidase-4 inhibitor; AMI, acute myocardial infarction.

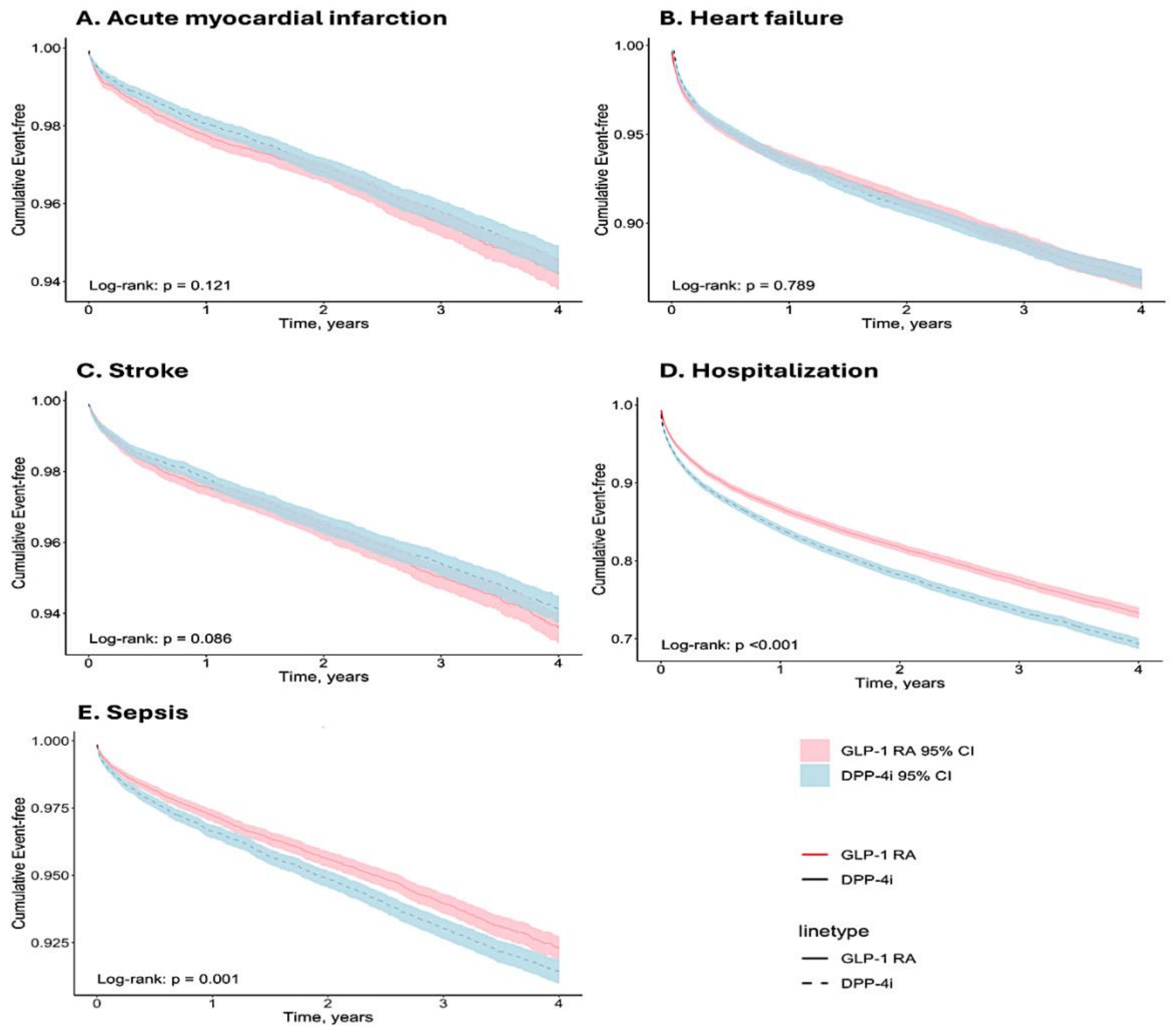

Figure S4. Subgroup Analyses for MAKE

Subgroup	GLP-1 RA events (%)	DPP4i events (%)	HR (95%CI)	p value	Heterogeneity p value
**Sex**					**0.779**
Male	1883(16.7)	2148(19)	0.92 (0.87 - 0.98)	0.011	
Female	1248(13.2)	1414(14.9)	0.94 (0.87 - 1.01)	0.085	
**Race**					**0.647**
White	1838(15.0)	2086(17.1)	0.93 (0.87 - 0.99)	0.023	
Africa American	562(18)	657(21.1)	0.88 (0.78 - 0.98)	0.02	
Asian	254(14.4)	291(16.5)	0.92 (0.78 - 1.09)	0.357	
**Age**					**0.048**
≥65	2015(19.6)	2187(21.2)	0.97 (0.91 - 1.03)	0.294	
18-64	1370(11.1)	1646(13.3)	0.88 (0.82 - 0.95)	<0.001	
**BMI (kg/m²)**					**0.853**
25-30	1503(13.3)	1743(15.4)	0.92 (0.86 - 0.99)	0.022	
<25	392(15.8)	471(19.0)	0.91 (0.80 - 1.04)	0.163	
**HbA1c(%)**					**0.002**
≥7	1015(14.5)	1048(15.0)	1.05 (0.97 - 1.15)	0.224	
<7	283(13.7)	387(18.8)	0.76 (0.65 - 0.88)	<0.001	
**eGFR (ml/min/1.73m²)**					**0.71**
≥60	864(11.3)	1000(13.0)	0.94 (0.85 - 1.02)	0.153	
<60	931(34.8)	1003(37.5)	0.96 (0.88 - 1.05)	0.36	
**IHD**					**0.539**
Yes	512(26.3)	591(30.3)	0.89 (0.79 - 1.00)	0.061	
No	2658(13.7)	3039(15.6)	0.93 (0.88 - 0.98)	0.006	
**HF**					**0.595**
Yes	287(45.9)	300(48.0)	0.94 (0.80 - 1.10)	0.419	
No	2847(13.7)	3361(16.2)	0.89 (0.85 - 0.94)	<0.001	

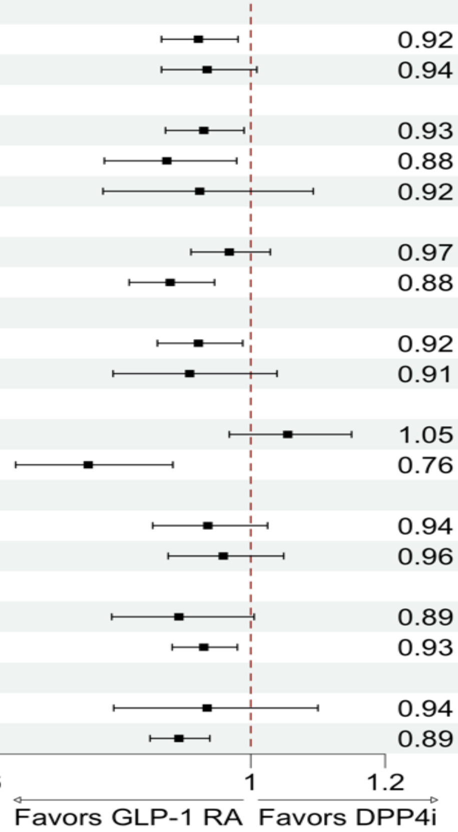

MAKE: GLP-1 RA vs. DPP4i

Abbreviations: MAKE, major adverse kidney events; GLP-1 RA, glucagon-like peptide-1 receptor agonist; DPP-4i, dipeptidyl peptidase-4 inhibitor; eGFR, estimated glomerular filtration rate; HF, heart failure; IHD, ischemic heart disease; HbA1c, glycated hemoglobin.

Figure S5. Subgroup Analyses for ESKD on Dialysis

Subgroup	GLP-1 RA events (%)	DPP4i events (%)	HR (95%CI)	p value	Heterogeneity p value
**Sex**					**0.386**
Male	370(3.3)	461(4.1)	0.84 (0.73 - 0.96)	0.01	
Female	228(2.4)	314(3.3)	0.76 (0.64 - 0.90)	0.001	
**Race**					**0.549**
White	268(2.2)	339(2.8)	0.83 (0.71 - 0.97)	0.022	
Africa American	129(4.1)	181(5.8)	0.73 (0.58 - 0.91)	0.006	
Asian	67(0.038)	97(5.5)	0.71 (0.52 - 0.98)	0.034	
**Age (years)**					**0.216**
≥65	316(3.1)	394(3.8)	0.84 (0.72 - 0.97)	0.019	
18-64	337(2.7)	477(3.9)	0.74 (0.64 - 0.85)	<0.001	
**BMI (kg/m²)**					**0.245**
25-30	287(2.5)	350(3.1)	0.87 (0.74 - 1.01)	0.074	
<25	79(3.2)	119(4.8)	0.71 (0.54 - 0.95)	0.021	
**HbA1c(%)**					**0.113**
≥7	164(2.3)	184(2.6)	0.97 (0.78 - 1.19)	0.751	
<7	65(3.2)	94(4.6)	0.71 (0.52 - 0.97)	0.033	
**eGFR (ml/min/1.73m²)**					**0.66**
≥60	50(0.7)	68(0.9)	0.82 (0.57 - 1.19)	0.293	
<60	243(9.1)	278(10.4)	0.90 (0.76 - 1.07)	0.232	
**IHD**					**0.851**
Yes	95(4.9)	114(5.9)	0.86 (0.65 - 1.13)	0.273	
No	508(2.6)	639(3.3)	0.83 (0.74 - 0.94)	0.002	
**HF**					**0.442**
Yes	72(11.5)	84(13.4)	0.85 (0.62 - 1.16)	0.297	
No	530(2.6)	744(3.6)	0.74 (0.66 - 0.83)	<0.001	

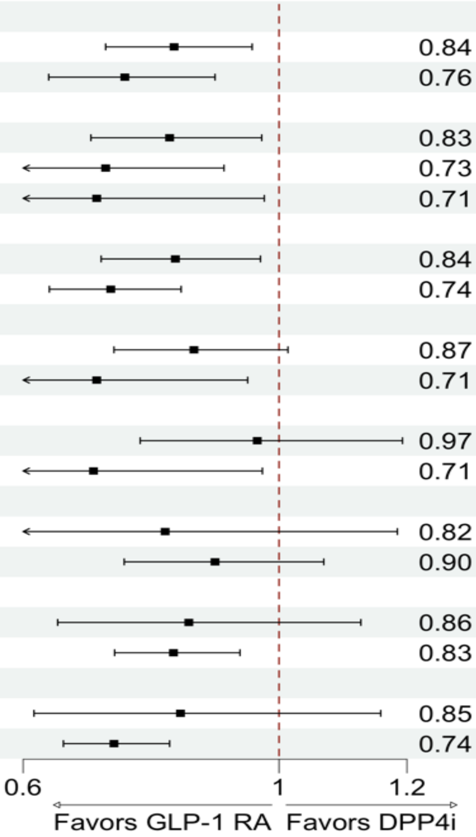

ESRD on dialysis: GLP-1 RA vs. DPP4i

Abbreviations: ESKD, end-stage kidney disease; GLP-1 RA, glucagon-like peptide-1 receptor agonist; DPP-4i, dipeptidyl peptidase-4 inhibitor; eGFR, estimated glomerular filtration rate; HF, heart failure; IHD, ischemic heart disease; HbA1c, glycated hemoglobin.

Figure S6. Distribution of Follow-up Time After Matching.

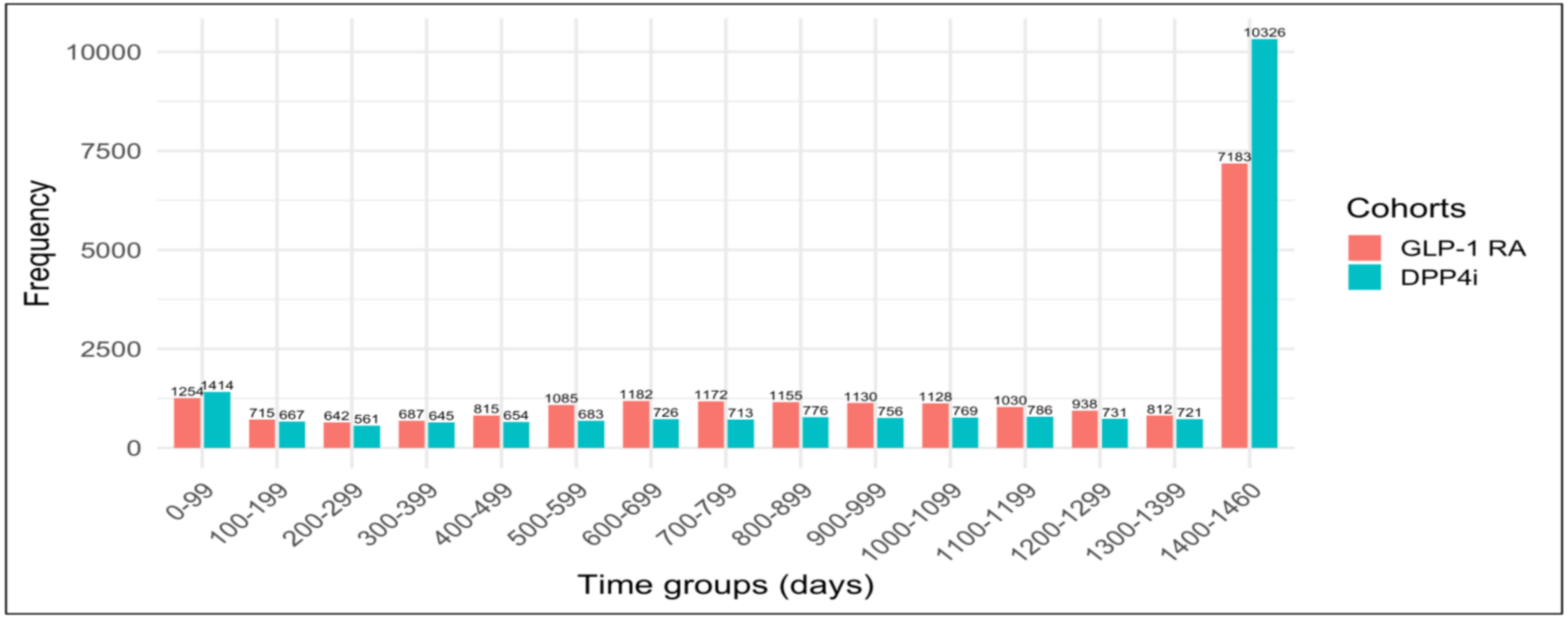

Follow-up Time (After Matching)

Cohort	Mean Follow-up (Days)	Standard Deviation	Median Follow-up (Days)	Interquartile Range
GLP-1 RA	968.050	476.472	1050	862.500
DPP4i	1059.494	496.834	1373	781

Abbreviations: GLP-1 RA, glucagon-like peptide-1 receptor agonist; DPP-4i, dipeptidyl peptidase-4 inhibitor.
